# Editorial: Role of complement activation in kidney diseases

**DOI:** 10.3389/fmed.2023.1297938

**Published:** 2023-10-19

**Authors:** Takashi Oda, Toshihiro Sawai

**Affiliations:** ^1^Department of Nephrology and Blood Purification, Kidney Disease Center, Tokyo Medical University Hachioji Medical Center, Hachioji, Japan; ^2^Department of Pediatrics, Shiga University of Medical Science, Otsu, Japan

**Keywords:** complement activation, alternative pathway, classical pathway, lectin pathway, kidney diseases, eculizumab, avacopan

The kidneys are often susceptible to complement activation-associated injury (1). Low serum complement levels resulting from complement activation are classically observed in kidney diseases, such as infection-related glomerulonephritis, membranoproliferative glomerulonephritis, and lupus nephritis. Thrombotic microangiopathy (TMA), including atypical hemolytic uremic syndrome (aHUS), is garnering a lot of attention as a disease caused by the aberrant activation of the alternative complement pathway. Complement activation contributes to disease pathogenesis, even in diseases without decreased serum complement levels, such as ANCA-associated glomerulonephritis (AAGN) (2).

Meanwhile, advancement has been made in the treatment of various diseases focusing on complement regulation since eculizumab, which blocks C5 of the complement pathway, was confirmed to be highly effective against aHUS (3). Molecular-targeted drugs in the field of complement regulation therapy are continuously being developed and promptly applied in clinical practice. Therefore, elucidation of the major complement activation pathways involved in tissue damage has directly led to the identification of therapeutic targets and the establishment of new therapeutic methods. Various medical agents that control specific points of complement activation, such as C3 (pegcetacopan), C5 (eculizumab), C5aR (avacopan), factor B (iptacopan), and factor D (danicopan), are being developed and applied clinically to treat certain pathogenic conditions (Figure 1).

**Figure 1:**
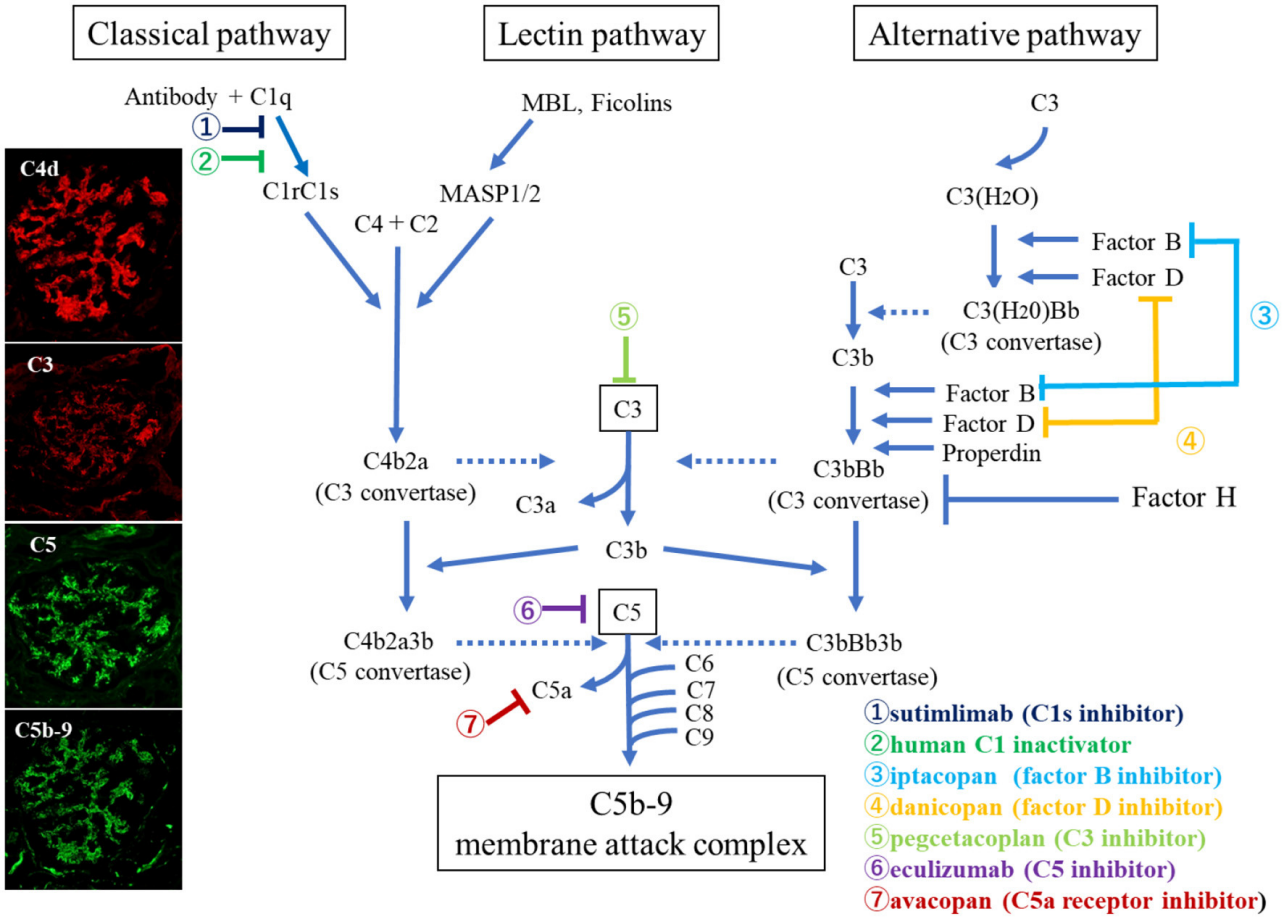
Overview of the complement activation system with therapeutic targets (13). Photographs on the left show glomerular deposition of complement breakdown products (C4d, C3, C5, and C5b-9) in a patient with ANCA-associated glomerulonephritis (AAGN), suggesting that classical pathway activation plays a role in the pathogenesis of AAGN.

In this Research Topic entitled “*Role of complement activation in kidney diseases*,” we published one case report, three original research articles, and one review article, all of which focus on this expanding topic of research.

Yamaguchi et al. described an extremely rare case of systemic lupus erythematosus (SLE) with both TMA and macrophage activation syndrome that was refractory to conventional therapy but responded well to eculizumab. Wright et al. reported the efficacy of eculizumab in the treatment of SLE-associated TMA (4). This significant response to eculizumab indicates the importance of the complement system in disease pathogenesis. They also discussed the possibility that serum syndecan-1 and hyaluronan levels may be useful biomarkers for detecting endothelial cell damage in such cases, as they change with disease status and response to treatment.

Two of the three original studies published on this Research Topic were related to the roles of complement activation in renal vascular lesions. Guo et al. paid special attention to a group of patients with IgA nephropathy manifesting rapid deterioration of renal function without hematuria and histological characteristics of intrarenal arteriolar lesions (5). They found significant deposition of complement components such as MBL, C4d, factor H, factor H-related protein 5, and C5b-9 on the walls of injured intrarenal arterioles, indicating the important role played by complement activation in the pathogenesis of arteriolar damage in this phenotype of patients with IgAN, leading to poor renal prognosis. Conversely, Chen et al. analyzed the deposition of complement components in renal biopsy tissues from arterionephrosclerosis based on previous studies that found the complement system to play an active role in the pathogenesis of hypertension and hypertension-related target organ damage (6, 7). They found significant deposition of C3d in the arterioles of renal biopsy tissues from patients with arterionephrosclerosis and a significant correlation between C3d deposition in the arterioles and macrophage infiltration around the vessels, thus confirming the potential role of complement activation in the pathogenesis of vascular lesions in arterionephrosclerosis.

The complement system is involved in peritoneal injury and protection during peritoneal dialysis (PD) (8–10). Kobayashi et al. analyzed membrane complement regulators (CRegs), such as CD46, CD55, and CD59, produced by primary cultured mesothelial cells (human peritoneal mesothelial cells; HPMCs), along with the levels of sC5b-9 in the PD fluids of patients. They found a significant decrease in sC5b-9 levels along with a significant increase in the expression of CD46 and CD59 over a period of 1 year. They also analyzed the effects of different PD fluids (glucose, lactic acid, and pH) on CReg expression in a human mesothelial cell line (Met-5A) *in vitro*. They found an increased expression of all three CRegs by glucose and lactic acid in a concentration-dependent manner; however, the expression of CRegs was conversely decreased by lower pH stimulation. Based on these results, the authors concluded that mesothelial cells may alter the expression of CRegs to protect the peritoneum and maintain peritoneal homeostasis via the complement system in patients with PD.

Kojima and Oda comprehensively summarized various reports on the role of complement activation in AAGN. In this field, detailed analyses have been conducted using animal models, and alternative pathways have been reported to be centrally involved. However, this has not been fully analyzed in human AAGN, and opinions are divided, with certain reports attributing primary involvement to the alternative pathway and others to the classical or lectin pathway (2, 11–14). Further studies are required to confirm this hypothesis. In the last part of the review, the results of a phase III clinical trial (ADVOCATE) on the therapeutic effect of a C5a receptor antagonist, avacopan, on ANCA-associated vasculitis (AAV) is presented (15). The results showed that avacopan is an alternative to glucocorticoids, has relatively few adverse effects, and is the first established anti-complement therapy available for use in the clinical field of AAV.

Therefore, elucidating the complement activation pathways involved in each renal disease is very important because it could unveil the targets of therapy for kidney diseases. Knowledge in this area is expanding and continued advancement is expected.

## Author contributions

TO: Conceptualization, Writing—original draft, Writing—review and editing. TS: Writing—review and editing.
